# Supplementary material for: Sexual dimorphism in obesity is governed by RELMα regulation of adipose macrophages and eosinophils
Source: bioRxiv. 2023 Jan 13:2023.01.13.523880. Preprint. [Version 1] doi: 10.1101/2023.01.13.523880 (PMC9882128; doi:10.1101/2023.01.13.523880)
Supplement: 1 [file NIHPP2023.01.13.523880v1-supplement-1.pdf]

bioRxiv preprint doi: https://doi.org/10.1101/2023.01.13.523880; this version posted January 13, 2023. The copyright holder for this preprint (which was not certified by peer review) is the author/funder, who has granted bioRxiv a license to display the preprint in perpetuity. It is made available under aCC-BY 4.0 International license.

Supplemental figures:

Figure S1. Flow cytometric analysis of adipose immune cells. (A-D) Flow cytometry analysis of the visceral adipose stromal vascular fraction of 12-week diet exposed mice for CD301 surface expression on $CD45^{+}CD64^{+}Mertk^{+}$ macrophages by tSNE plot and mean fluorescent intensity (MFI) **(A)** and surface expression by $CD45^{+}SiglecF^{+}CD11b^{+}$ eosinophils of CXCR4 **(B)**, MHCII **(C)** and F4/80 **(D)**. **(E-F)** Visceral fat leukocytes from naïve or *H. polyrus*-infected mice were quantified **(E)**, and eosinophils were column purified and analyzed for purity by flow and cytocentrifuge **(F)**. Statistical significance was determined by unpaired t-test (*, $p < 0.05$; **, $p<0.01$). tSNE plot analysis and flow plots are representative of one animal per group, all other data are presented as individual points for each animal, where lines represent group means +/- S.E.M. Statistical significance for (A-D) was determined by two-way ANOVA with Sidak's multiple comparisons test. (ns, no significant; *, $p < 0.05$; **, $p<0.01$; ***, $p<0.001$) and by unpaired t-test for (E). Data are representative of two experiments with 3-6 mice per group.

Figure S2. Differentially expressed genes in fibroblast and ILC2 cell populations from scRNA-seq analysis. (A) Gene ontology (GO) pathways of the top 75 genes that were upregulated in WT females vs other three group in all clusters. **(B)** Pathway network analysis of the top 75 genes upregulated in WT females vs other three groups. **(C)** Population-shift in fibroblasts between WT female, WT male, KO female and KO male. **(D-L)** Differentially expressed genes in fibroblast population between WT female vs. WT male, WT female vs KO female, WT male vs KO male as associated GO terms for top DEG. **(M-P)** ILC2 cell clusters and

bioRxiv preprint doi: https://doi.org/10.1101/2023.01.13.523880; this version posted January 13, 2023. The copyright holder for this preprint (which was not certified by peer review) is the author/funder, who has granted bioRxiv a license to display the preprint in perpetuity. It is made available under aCC-BY 4.0 International license.

DEG between WT females and WT males. Heatmaps indicate DEGs between WT female, WT male, KO female and KO males.

Figure S3. **Gene ontology pathway analysis for the top 30 hits in myeloid cell subsets.**

(A) GO pathway analyses comparing the top 30 genes between WT female vs WT male, and KO female vs KO male in myeloid cell subsets. **(B)**: GO comparing the top 30 genes between WT female vs KO female and WT male vs KO male in myeloid cell subsets

Figure S4. **Monocyte and Mac1 DEG**

(A) GO pathway analyses of top 30 DEG in WT female monocytes and Mac1 compared to other groups. **(B)** Heatmaps indicating DEGs in Monocyte and Mac1 and average UMI count analysis between all groups for key marker genes. **(C)** WT female vs KO female on HFD monocyte subcluster DEG presented as Log2 fold changes in a heatmap and average UMI count comparisons.

Table 1. Three-way ANOVA analysis of Body weight, Immune cells, Macrophages and Eosinophils.

(which was not certified by peer review) is the author/funder, who has granted bioRxiv a license to display the preprint in perpetuity. It is m
available under aCC-BY 4.0 International license.

Supplemental Figure 1.

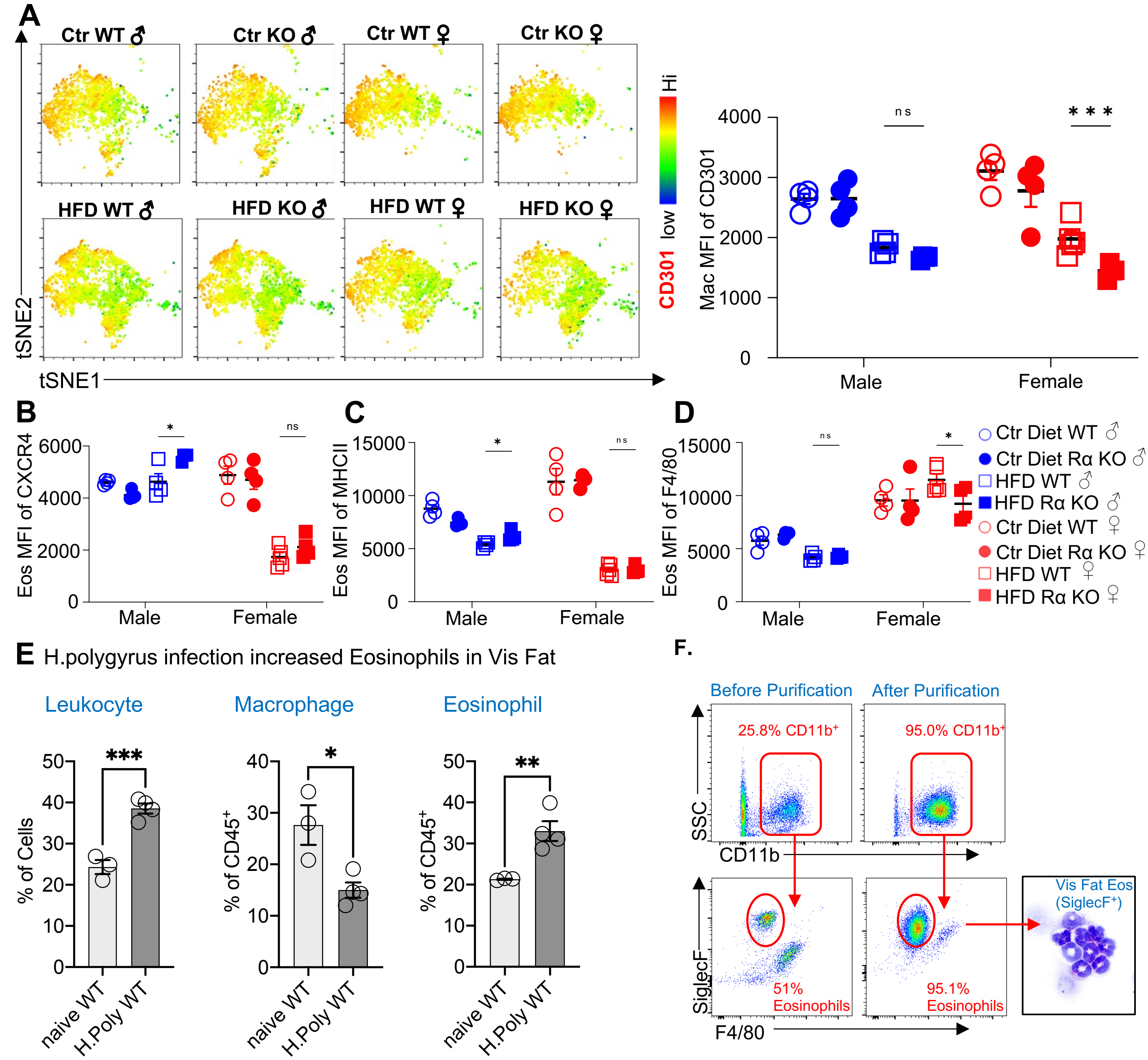

(which was not certified by peer review) is the author/funder, who has granted bioRxiv a license to display the preprint in perpetuity. It is made available under aCC-BY 4.0 International license.

Supplemental Figure 2.

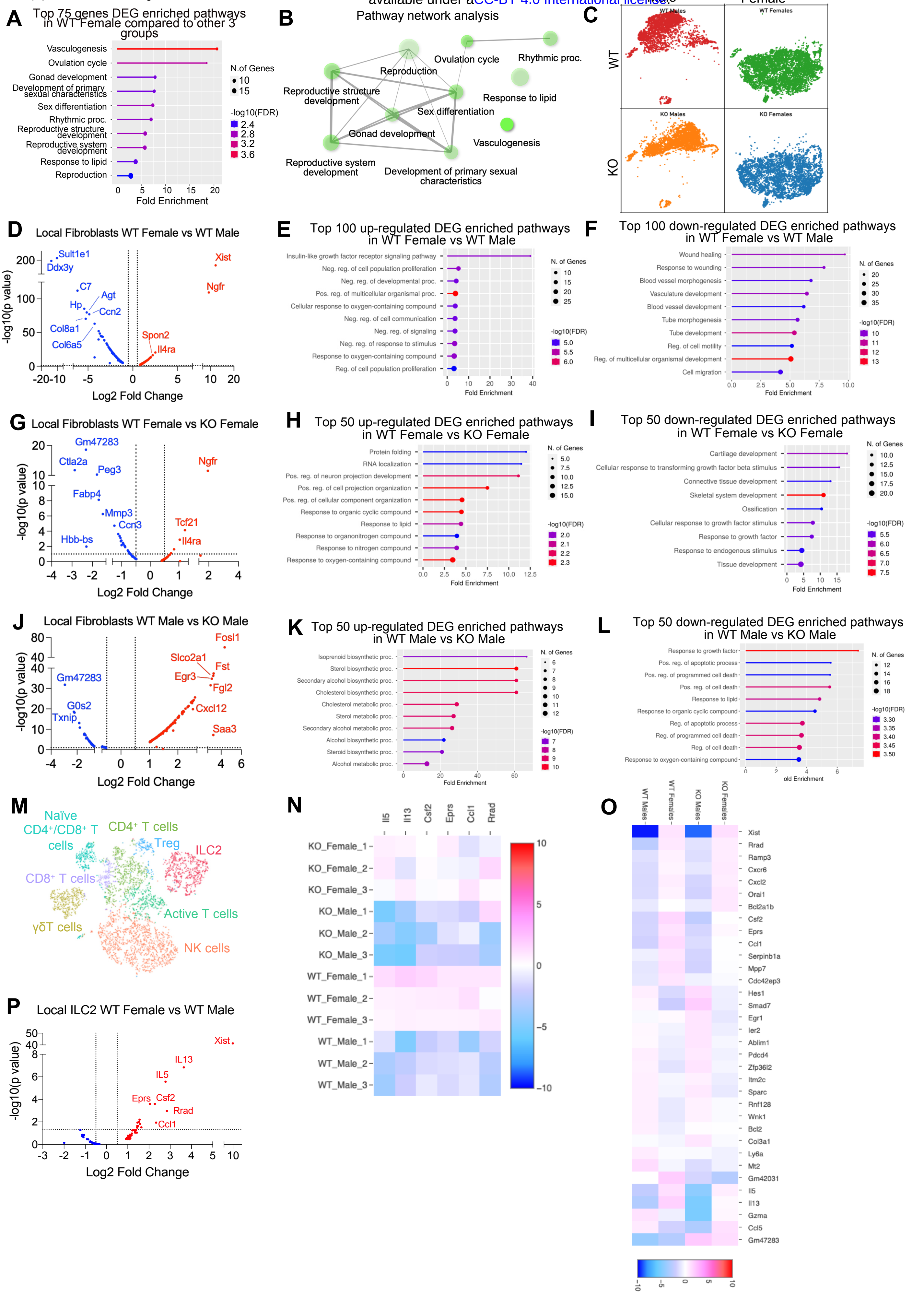

(which was not certified by peer review) is the author/funder, who has granted bioRxiv a license to display the preprint in perpetuity. It is m
available under aCC-BY 4.0 International license.

Supplemental Figure 3.

Myeloid cells (pathway analysis for top 30 hits):

A

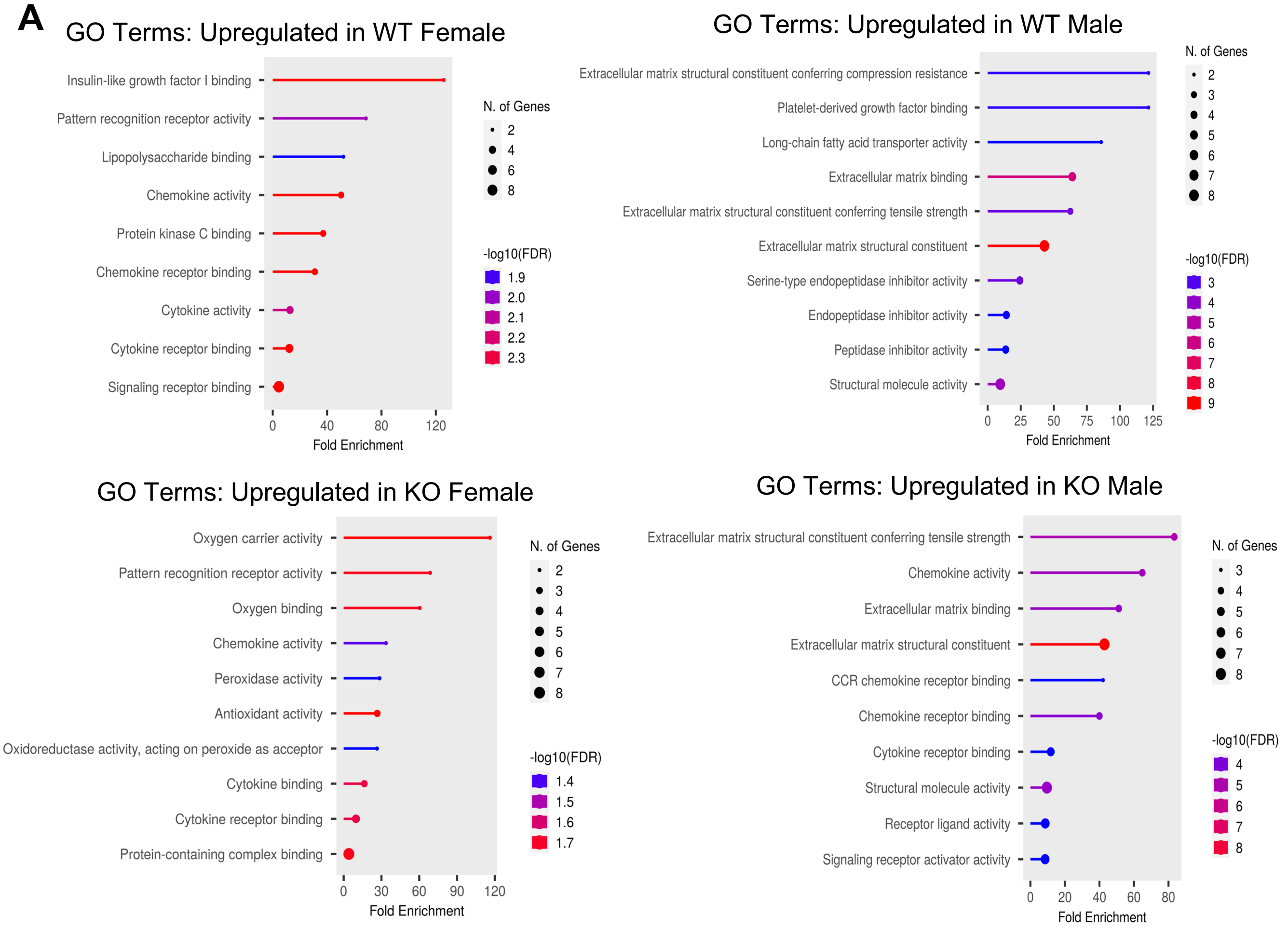

B

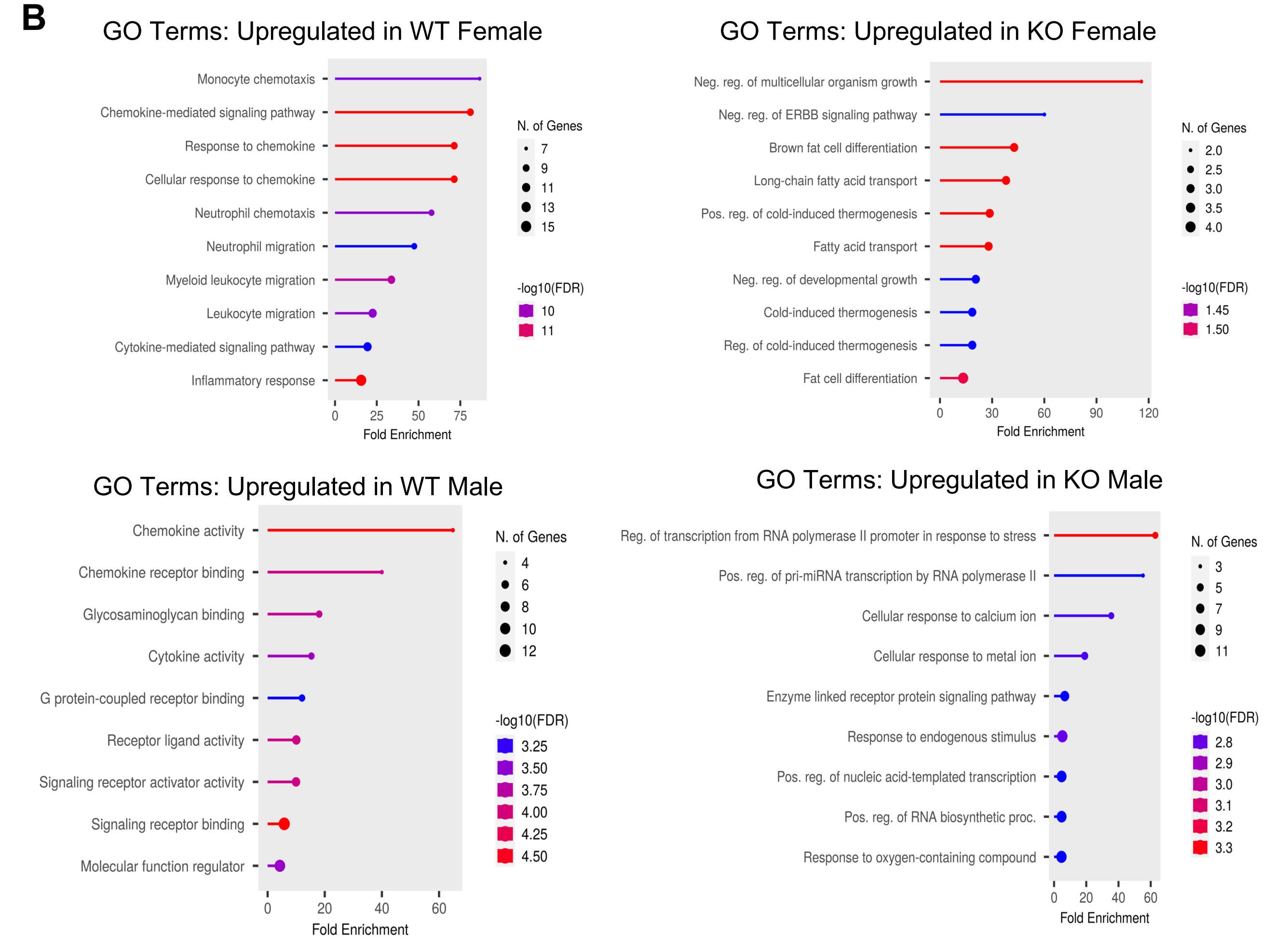

(which was not certified by peer review) is the author/funder, who has granted bioRxiv a license to display the preprint in perpetuity. It is m
available under aCC-BY 4.0 International license.

Supplemental Figure 4.

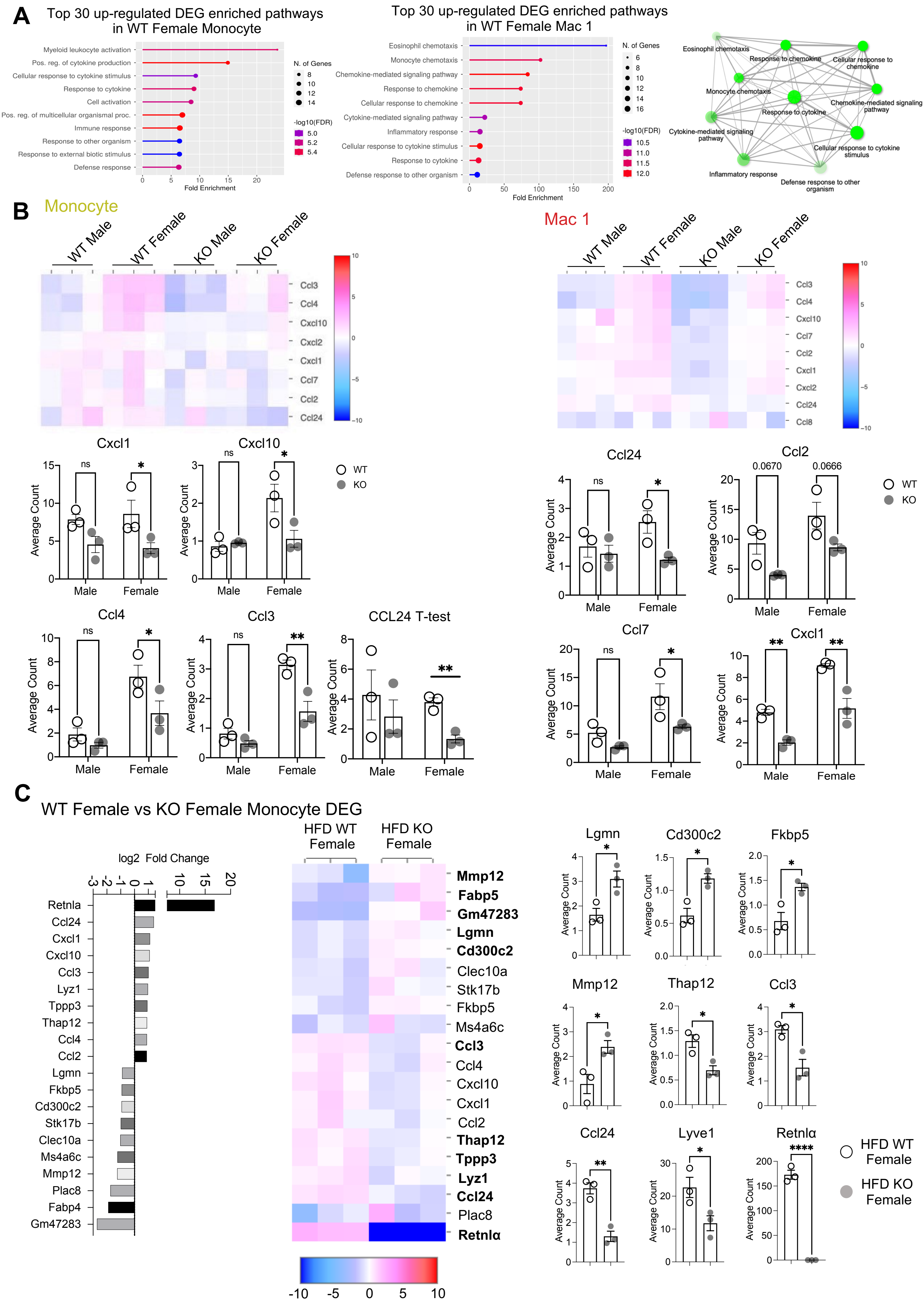

(which was not certified by peer review) is the author/funder, who has granted bioRxiv a license to display the preprint in perpetuity. It is m
available under aCC-BY 4.0 International license.

Supplemental Table 1.

Mouse body weight 6 weeks Post Diet **3Way ANOVA**

Source of Variation	% of total variation	P value	P value summary	Significant?
Male Vs Female	34.40	<0.0001	****	Yes
Ctr Vs HFD	34.04	<0.0001	****	Yes
WT Vs KO	4.226	0.0056	**	Yes
Male Vs Female x Ctr Vs HFD	5.005	0.0028	**	Yes
Male Vs Female x WT Vs KO	2.019	0.0484	*	Yes
Ctr Vs HFD x WT Vs KO	0.6178	0.2651	ns	No
Male Vs Female x Ctr Vs HFD x WT Vs KO	3.861	0.0078	**	Yes

Mouse body weight 12 weeks Post Diet **3Way ANOVA**

Source of Variation	% of total variation	P value	P value summary	Significant?
Male Vs Female	13.13	<0.0001	****	Yes
Ctr Vs HFD	71.83	<0.0001	****	Yes
WT Vs KO	3.335	0.0003	***	Yes
Male Vs Female x Ctr Vs HFD	1.000	0.0319	*	Yes
Male Vs Female x WT Vs KO	1.668	0.0067	**	Yes
Ctr Vs HFD x WT Vs KO	2.663	0.0009	***	Yes
Male Vs Female x Ctr Vs HFD x WT Vs KO	1.668	0.0067	**	Yes

Leukocytes in % of SVF **3Way ANOVA**

Source of Variation	% of total variation	P value	P value summary	Significant?
Male Vs Female	9.708	<0.0001	****	Yes
Ctr Vs HFD	55.05	<0.0001	****	Yes
WT Vs KO	17.48	<0.0001	****	Yes
Male Vs Female x Ctr Vs HFD	3.345	0.0043	**	Yes
Male Vs Female x WT Vs KO	0.005690	0.8980	ns	No
Ctr Vs HFD x WT Vs KO	0.3134	0.3459	ns	No
Male Vs Female x Ctr Vs HFD x WT Vs KO	7.520	<0.0001	****	Yes

Macrophage in % of Leukocytes **3Way ANOVA**

Source of Variation	% of total variation	P value	P value summary	Significant?
Male VS Female	8.384	0.0068	**	Yes
Ctr Vs HFD	17.40	0.0003	***	Yes
WT Vs KO	2.665	0.1084	ns	No
Male VS Female x Ctr Vs HFD	38.38	<0.0001	****	Yes
Male VS Female x WT Vs KO	0.6558	0.4167	ns	No
Ctr Vs HFD x WT Vs KO	7.337	0.0106	*	Yes
Male VS Female x Ctr Vs HFD x WT Vs KO	0.0003325	0.9853	ns	No

Eosinophils in % of Leukocytes **3Way ANOVA**

Source of Variation	% of total variation	P value	P value summary	Significant?
Male Vs Female	54.11	<0.0001	****	Yes
Ctr Vs HFD	19.61	<0.0001	****	Yes
WT Vs KO	1.364	0.1743	ns	No
Male Vs Female x Ctr Vs HFD	2.298	0.0815	ns	No
Male Vs Female x WT Vs KO	0.001983	0.9579	ns	No
Ctr Vs HFD x WT Vs KO	1.396	0.1695	ns	No
Male Vs Female x Ctr Vs HFD x WT Vs KO	3.595	0.0321	*	Yes
